# Promoter de-methylation of *cyclin D2 *by sulforaphane in prostate cancer cells

**DOI:** 10.1186/1868-7083-3-3

**Published:** 2011-10-26

**Authors:** Anna Hsu, Carmen P Wong, Zhen Yu, David E Williams, Roderick H Dashwood, Emily Ho

**Affiliations:** 1 School of Biological and Population Health Sciences, 103 Milam Hall, Oregon State University, Corvallis, OR 97331, USA; 2 Linus Pauling Institute, 571 Weniger Hall, Oregon State University, Corvallis, OR 97331, USA; 3 Department of Environmental & Molecular Toxicology, 1007 Ag Life Sciences Building, Oregon State University, Corvallis, OR 97331, USA

**Keywords:** Sulforaphane, methylation, prostate cancer, cyclins, chemo-prevention

## Abstract

Sulforaphane (SFN), an isothiocyanate derived from cruciferous vegetables, induces potent anti-proliferative effects in prostate cancer cells. One mechanism that may contribute to the anti-proliferative effects of SFN is the modulation of epigenetic marks, such as inhibition of histone deacetylase (HDAC) enzymes. However, the effects of SFN on other common epigenetic marks such as DNA methylation are understudied. Promoter hyper-methylation of *cyclin D2*, a major regulator of cell cycle, is correlated with prostate cancer progression, and restoration of *cyclin D2 *expression exerts anti-proliferative effects on LnCap prostate cancer cells. Our study aimed to investigate the effects of SFN on DNA methylation status of *cyclin D2 *promoter, and how alteration in promoter methylation impacts *cyclin D2 *gene expression in LnCap cells. We found that SFN significantly decreased the expression of DNA methyltransferases (DNMTs), especially *DNMT1 *and *DNMT3b*. Furthermore, SFN significantly decreased methylation in *cyclin D2 *promoter regions containing c-Myc and multiple Sp1 binding sites. Reduced methlyation of *cyclin D2 *promoter corresponded to an increase in *cyclin D2 *transcript levels, suggesting that SFN may de-repress methylation-silenced *cyclin D2 *by impacting epigenetic pathways. Our results demonstrated the ability of SFN to epigenetically modulate *cyclin D2 *expression, and provide novel insights into the mechanisms by which SFN may regulate gene expression as a prostate cancer chemopreventive agent.

## Introduction

Studies have shown that high consumption of cruciferous vegetables is inversely associated with prostate cancer risk [1-5]. Cruciferous vegetables and their biologically active constituents, including isothiocyanates (ITCs) such as sulforaphane (SFN), appear to modulate prostate cancer risk at multiple stages of carcinogenesis. SFN is an effective chemoprotective agent for prostate cancer in both *in vitro *and *in vivo *models by selectively inducing apoptosis and slowing tumor growth [6-10]. More recently, SFN has been shown to induce anti-proliferative effects via epigenetics, namely acting as a dietary histone deacetylase (HDAC) inhibitor. SFN treatment leads to an increase in histone acetylation and re-expression of various tumor suppressor genes [11-13]. SFN-mediated epigenetic alterations may not only be limited to HDAC regulation. Studies suggest that SFN may play an important role in methyl CpG-binding proteins' recruitment of HDAC family members [14]. In breast cancer cells, SFN suppresses DNA methylation in the *hTERT *promoter, leading to transcriptional repression [15]. Together, these data suggest that there may be multiple mechanisms by which SFN epigenetically regulates gene expressions.

Cell cycle progression is controlled by cyclin-dependent kinases (CDKs) and their activities are further regulated by cyclins and CDK inhibitors. D-type cyclins (D1, D2, and D3) are mainly implicated in G1 to S phase transition [16]. Dys-regulation of the cyclin Ds disrupts cell cycle control and promotes neoplastic transformation. *Cyclin D2/CCND2 *has been identified in several cancers as a proto-oncogene. Over-expression of *cyclin D2 *correlates with progression and poor prognosis in gastric cancer [17,18], colon cancer [19] and granulosa cell tumors of the ovary [20]. However, silencing of *cyclin D2 *expression by promoter methylation is also associated with cancer progression in breast cancer [21-23], lung [23], pancreatic [24] and gastric cancer [25], suggesting that *cyclin D2 *might act as a tumor suppressor gene in a cancer-type dependent manner. In prostate cancer, increased *cyclin D2 *promoter methylation corresponds to a decrease in *cyclin D2 *mRNA expression, and correlates with higher Gleason scores and pathologic features of tumor aggressiveness [26]. The present study was undertaken to evaluate the effects of SFN on the epigenetic regulation of *cyclin D2 *in prostate cancer cells. Our results indicate that SFN may down-regulate DNA methyltransferases (DNMTs) resulting in de-methylation of the *cyclin D2 *promoter and de-repression of *cyclin D2 *expression, and suggest a novel mechanism behind SFN's growth inhibitory effects on prostate cancer cells.

## Materials and methods

### Cell Culture and Treatment

Benign prostate hyperplasia (BPH-1) cells were generously donated by Dr. Simon Hayward (Vanderbilt University Medical Center, Nashville, TN); androgen dependent prostate cancer epithelial cells (LnCap) and androgen-independent prostate cancer epithelial cells (PC3) were obtained from America Type Culture Collection (Manassas, VA). Cells were grown and maintained in RPMI 1640 supplemented with glutamine (Mediatech Inc., Manassas, VA), 1% penicillin-streptomycin (Mediatech Inc., Manassas, VA) and 10% (5% for BPH-1 cells) fetal bovine serum (FBS) (Hyclone, Logan, UT) under standard conditions (5% CO_2 _, 37°C, humidified atmosphere). R, S-Sulforaphane (SFN, LKT Laboratories, Inc., St. Paul, MN) was dissolved in dimethyl sulfoxide (DMSO) or ethanol (EtOH) (Sigma-Aldrich). Vehicle treatments consisted of DMSO or EtOH in a final concentration matching the highest level of treated cells (30 μM SFN) and was less than 0.1% of the final volume. Cells were treated with vehicle or SFN (15 or 30 μM) for 24 or 48 hours.

### mRNA expression levels of DNMTs by quantitative real-time PCR (qRT-PCR)

Total RNA was extracted using TRIzol Reagent (Invitrogen, Carlsbad, CA) according to the manufacturer's instructions. Total RNA (1 μg) was reverse-transcribed into cDNA using SuperScript III First-Strand Synthesis Supermix (Invitrogen, Carlsbad, CA) containing reaction buffer, MgCl_2_, dNTPs and a 1:1 mixture of random hexamers and oligo-dT primers. For qRT-PCR, the primer sequences were as follows: *DNMT1*, (sense) 5'GTGGGGGACTGTGTCTCTGT-3' and (antisense) 5'-TGAAAGCTGCATGTCCTCAC-3'; *DNMT3a*, (sense) 5'-CACACAGAAGCATATCCAGGAGTG-3' and (antisense) 5'-AGTGGACTGGGAAACCAAATACCC-3'; *DNMT3b*, (sense) 5'-AATGTGAATCCAGCCAGGAAAGGC-3' and (antisense) 5'-ACTGGATTACACTCCAGGAACCGT-3'; *CCND2*, (sense) 5'-TGGAGCTGCTGTGCCACG -3' and (antisense) 5'-GTGGCCACCATTCTGCGC-3' and *GAPDH *(sense) 5'- CGAGATCCCTCCAAAATCAA- 3' and (antisense) 5'-TTCACACCCATGACGAACAT-3'. The reactions were performed using DyNAmo HS SYBRGreen qPCR kit (New England Biolabs, Ipswich, MA) on a Chromo4 Thermal Cycler (MJ Research, Inc., Waltham, MA). PCR conditions were programmed as follows: 95°C for 5 min, followed by 40 cycles of denaturing at 95°C for 10 s, annealing at 60°C (62°C for *CCND2*) for 20 s, and extension at 72°C for 20 s. A dilution series of 10^2^, 10^3^, 10^4^, 10^5^, and 10^6 ^copies of template DNA served as internal standard for quantification. Gene copy numbers were calculated based on standard curves using Opticon Monitor 2 Software (MJ Research, Inc.) and normalized to *GAPDH*. Data represent normalized fold-change +/- SEM.

### Western blot analysis

Whole cell lysates were isolated by lysing cells with immunoprecipitation (IP) buffer (25 mM HEPES, pH 7.4; 150 mM NaCl; 1 mM EDTA; 0.5% Triton X-100) followed by flash freeze/thaw treatment and centrifugation. Protein concentrations were determined by the Bradford assay (Bio-Rad Protein Assay, Bio-Rad, Hercules, CA). Proteins (20-30 μg) were separated by SDS-PAGE on a 4-12% bis-Tris gel (NuPAGE Novex, Invitrogen, Carlsbad, CA) and transferred to nitrocellulose membranes (Invitrogen). Ponceau S red staining and β-actin protein levels were used as protein loading controls. Membranes were incubated in primary antibodies specific against *DNMT1 *(1:1000) (Santa Cruz Biotechnology, Inc., Santa Cruz, CA) overnight at 4°C. Secondary antibodies were conjugated with horseradish peroxidase (HRP) (Santa Cruz), and proteins were detected by Western Lightning Chemiluminescence Reagent Plus (PerkinElmer Life Sciences, Boston, MA) and imaged by Alpha Innotech photodocumentation system. Densitometry and quantifications were performed using NIH Image J software.

### Bisulfite modification

Genomic DNA from cells was extracted using the DNeasy Blood and Tissue Kit (Qiagen, Valencia, CA). Genomic DNA (0.5-1 μg) was treated with sodium bisulfite via the EZ DNA Methylation-Gold Kit (Zymo Research, Irvine, CA). Bisulfite treatments changed unmethylated cytosines into uracils while leaving methylated cytosines unmodified. Bisulfite-treated genomic DNA was used for further analysis of methylation status of CpG sites via methyl-specific PCR (MSP) and bisulfite DNA sequencing.

### Methyl-specific PCR (MSP)

PCR amplification was performed on the bisulfite--treated DNA samples using primer sets targeting CpG-rich region in the *cyclin D2 *promoter. The MSP primer sequences were based on previous reports and primer binding positions were numbered from the transcriptional start site [25]: unmethylated reaction, 5'- GTTATGTTATGTTTGTTGTATG -3' (sense, -1372 to -1350), and 5'- TAAAATCCACCAACACAATCA -3' (antisense, -1150 to -1170) and methylated reaction, 5'- GTTACGTTATGTTCGTTGTACG -3' (sense, -1372 to -1350), and 5'- TAAAATCGCCGCCAACACGATCG -3' (antisense, -1150 to -1173). PCR was conducted using MSP buffer containing 16.6 mM ammonium sulfate, 67 mM Tris (pH 8.8), 6.7 mM MgCl_2_, and 10 mM β-mercaptoethanol. Touchdown PCR condition was as follows: 95°C for 5 min, followed by 10 cycles of 95°C for 30 s, 62°C for 30 s, 72°C for 45 s, and 30 cycles of 95°C for 30 s, 55°C for 30 s, 72°C for 45 s, and a final extension step of 5 min at 72°C. PCR products (223-bp) were analyzed on 3% low melt agarose gels and stained with ethidium bromide.

### Bisulfite DNA sequencing

For bisulfite DNA sequencing analysis, genomic DNA was digested with AflIII restriction enzyme (New England Biolabs) and precipitated in the presence of GenElute LPA (Sigma-Aldrich, St. Louis, MO) prior to sodium bisulfite treatment. PCR primers were designed to amplify a CpG-rich region spanning from -1700 to -1250 bp from the transcription start site, which contains 24 CpG sites. Bisulfite primer sequences were: 5'-AGCTATTGGCTATGCAAATAGAGGG-3' (-1670 to -1645, forward) and 5'- CCCTTTAATATATTTCACTCCAA-3' (-1261 to -1284, reverse). PCR conditions described in the preceding section were used for amplification. Following PCR amplification, gel-purified bands were cloned into the pCR2.1 vector using the TOPO TA Cloning Kit (Invitrogen). Multiple clones (minimum of ten) from each PCR product were submitted for DNA sequencing at the Center for Genome Research & Biocomputing Core Laboratories (Oregon State University, Corvallis, OR). Transcription Element Search Software (TESS) accessed through the World Wide Web was used to identify transcription factor binding sites within the *cyclin D2 *promoter region.

### Global Methylation Status

Global methylation status of LnCap after treatments was assessed by using the MethylFlash™ Methylated DNA Quantification Kit (Epigentek, Brooklyn, NY) according to manufacturer's protocols with 200 ng of genomic DNA.

### Statistics

Data from independent triplicate (n = 3) experiments were collected and statistical significance between SFN-treated and other treatments were determined by one-way ANOVA and Tukey-Krammer Multiple Comparison test using GraphPad Prism V5.0 software (GraphPad, San Diego, CA). A P value of < 0.05 was considered statistically significant.

## Results

### Sulforaphane decreased DNA methyltransferase expression

**Figure 1:**
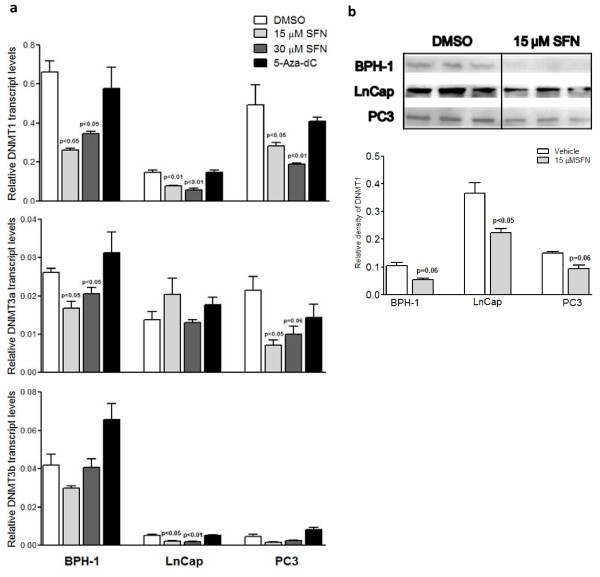
**SFN decreased mRNA and protein expressions of DNA methyltransferases in prostate cancer cells**. **a**. Benign hyperplasia cells (BPH-1), LnCap and PC3 cells were treated with SFN (15 and 30 μM) or 5-Aza-dC (5 μM) for 48 h. RNA was extracted and cDNA synthesized for qRT-PCR. Values are fold change of gene copy numbers normalized to *GAPDH *and compared to vehicle. **b**. Whole cell lysates were extracted and *DNMT1 *protein levels were analyzed by western blot. The lower graph represents the relative density of *DNMT1 *normalized to β-actin levels. Results represent means ± SEM, n = 3. P values represent treatment values compared to vehicle.

We assessed the effects of SFN on the expressions of DNMTs (*DNMT1, DNMT3a*, and *DNMT3b*) in benign hyperplasia (BPH-1), LnCap and PC3 prostate cancer cells. Cells were treated with SFN (15 μM and 30 μM), and assayed for *DNMT1, 3a *and *3b *transcript levels after 48 h. *DNMT1 *protein expression was also examined by western blot in treated cells. In BPH-1 and PC3 cells, SFN at both treatment doses significantly decreased *DNMT1 *and *3a *mRNA expression, but did not significantly change *DNMT1 *protein expression (Figure 1). In LnCap cells, SFN significantly decreased *DNMT1 *and *3b *mRNA expressions, and *DNMT1 *protein expression (Figure 1). Previous study indicated that concentrations around 10 μM SFN effectively inhibited HDAC activity in colonic mucosa *in vivo*, and one would consume about 1 cup (106 g) of broccoli sprouts per day to achieve similar plasma levels in humans [27]. This shows that the lowest treatment concentration in this study (15 μM) that decreased DNMT expressions was within practical limits. 5-Aza-dC did not significantly affect DNMTs expression, indicating that 5-Aza-dC inhibits DNMT enzymatic activity rather than expression, consistent with the mechanism by which 5-Aza-dC inhibits DNMT through direct binding [28]. Alternatively, global inhibition of DNA methylation by 5-Aza-dC treatments may result in AP-1 dependent induction of *DNMT1 *expression similarly to observation in mouse embryonal cell line, P19 [29]. SFN appeared to most significantly suppress transcript and protein levels of DNMTs in LnCap cells. Previous studies also demonstrated that over-expression of *cyclin D2 *induced anti-proliferative effects on LnCap cells, but not PC3 cells [30]. Therefore, LnCap cells were used to study subsequent endpoints.

### Sulforaphane decreased global DNA methylation and Cyclin D2 promoter methylation

**Figure 2:**
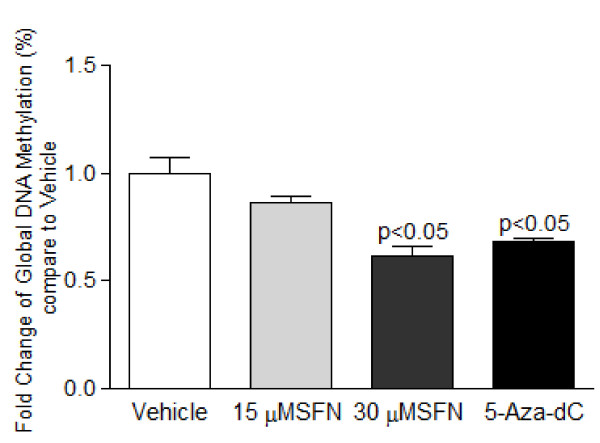
**Sulforaphane decreased global DNA methylation**. LnCap cells were treated with SFN (15 and 30 μM) or 5-Aza-dC (5 μM) for 24 h. Genomic DNA was extracted and treated with sodium bisulfite. Global methylation status was fluorometrically quantified in a microplate-based ELISA assay. Values were derived as fold change compared to vehicle (DMSO) and represent means ± SEM, n = 3. P values represent treatment values compared to vehicle.

DNA methylation plays an important role in regulating *cyclin D2 *expression ([20-23,25,26,31]. To explore the effects of SFN on DNA methylation, we first examined the effects of SFN treatment on the global methylation status of LnCap cells, followed by detailed investigation of the methylation status of *cyclin D2 *promoter region via methyl-specific PCR (MSP) and bisulfite sequencing analysis. SFN (30 μM) and 5-Aza-dC significantly decreased global methylation in LnCap cells after 24 h treatments (Figure 2), suggesting systematic de-methylating effects by SFN.

**Figure 3:**
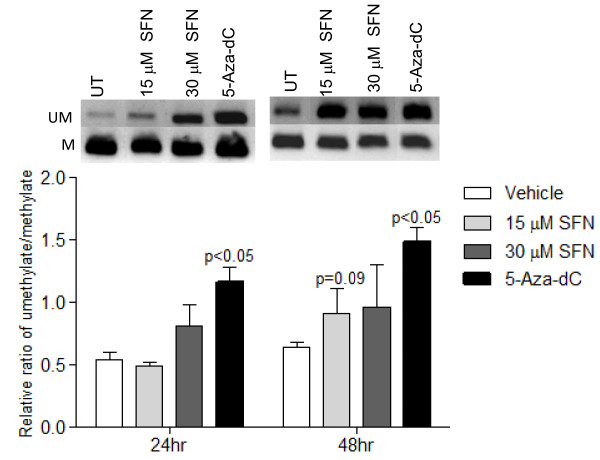
**Sulforaphane decreased promoter methylation of *cyclin D2***. Methylation-specific PCR was performed after bisulfite modification of DNA from LnCap cells treated for 48 h. Upper figures are representative bands. UM indicates unmethylated *cyclin D2 *PCR products. M indicates methylated PCR products. The lower figure shows the levels of unmethylated versus methylated *cyclin D2 *PCR products by densitometry based on biological triplicates. SFN and 5-Aza-dC both decreased *cyclin D2 *promoter methylation. P values represent treatment values compared to vehicle.

**Figure 4:**
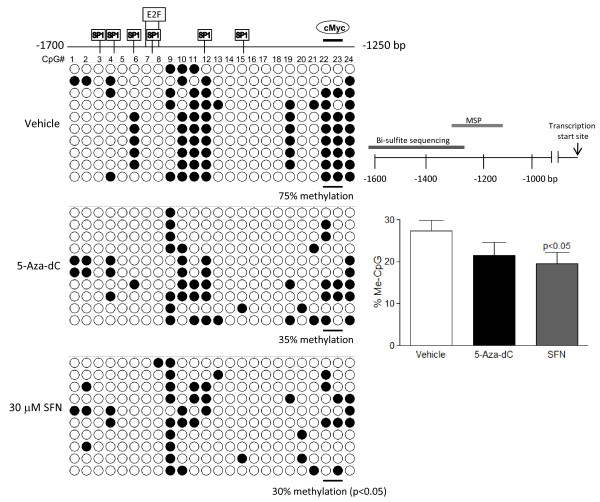
**Bisulfite sequencing of *cyclin D2 *promoter region**. The nucleotide sequence -1700 to -1250 bp from the transcription start site of the *cyclin D2 *gene promoter for the bisulfite sequencing analysis, and the relative regions of amplifications by MSP and bisulfite sequencing are shown. Genomic DNA from LnCap cells treated for 48 h was sodium bisulfite-treated, PCR amplified and subcloned. The sequencing results from 15-20 clones were used for analysis (upper figure, p values represent treatment values compared to vehicle), and representative 10 clones are shown in the dot plot. Each horizontal line represents the sequencing result of one subclone. Methylated CpG sites are shown as solid circles, whereas open circles indicate unmethylated CpG sites.

To specifically investigate the promoter methylation status of *cyclin D2*, an initial screen for *cyclin D2 *promoter methylation was performed by MSP, followed by verification via bisulfite sequence analysis. MSP screening suggested that there was a trend of decreasing methylation associated with increasing SFN treatment (Figure 3). Treatment with de-methylating agent, 5-Aza-dC, significantly increased un-methylated product compared to control. Since MSP is limited to provide semi-quantitative analysis of a few CpG sites; we verified the MSP findings with bisulfite sequencing. To study the extent of promoter methylation, the CpG-rich region of the *cyclin D2 *promoter further upstream spanning nucleotides -1700 to -1250 bp (a total of 24 CpG sites) was sequenced after bisulfite modification of genomic DNA from cells treated with SFN for 48 h. As shown in Figure 4, the CpG island examined was partially methylated in LnCap cells in the vehicle control group. 5-Aza-dC treatment produced a trend toward decreased methylation, and SFN-treatment resulted in a significant decrease in methylated CpG sites in this region. In particular, the c-Myc transcription factor binding site at CpG residue 23 and 24 were hyper-methylated in vehicle control, and SFN treatment significantly decreased methylation at these CpG sites (p < 0.05).

### Sulforaphane induced *cyclin D2 *expression in LnCap Cells

**Figure 5:**
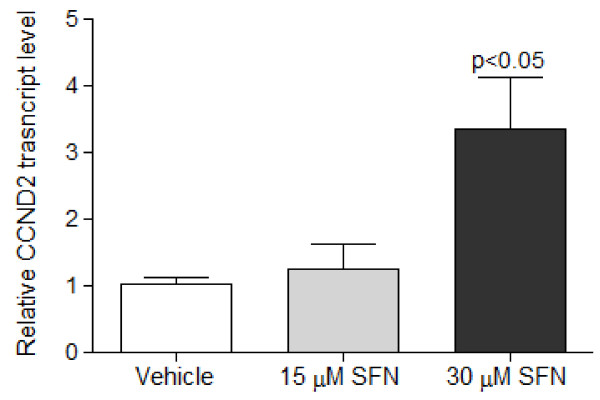
**Sulforaphane increased transcript levels of *cyclin D2***. LnCap cells were treated with SFN (15 and 30 μM) for 24 h. RNA was extracted and cDNA synthesized for qRT-PCR. SFN increased transcript levels of *cyclin D2 *compared with vehicle (DMSO). Values are fold change of gene copy numbers normalized to *GAPDH *and compared to vehicle, and represent means ± SEM, n = 3. P values represent treatment values compared to vehicle.

Previous reports stated that promoter methylation of *cyclin D2 *was associated with transcriptional silencing in gastric cancer cell lines [25]. By using qRT-PCR, *cyclin D2 *mRNA expression was determined in prostate cancer cells treated with SFN. At 24 h, SFN dose-dependently increased *cyclin D2 *mRNA expression (Figure 5). There were no significant changes in *cyclin D2 *mRNA levels associated with 5-Aza-dC treatment (data not shown), possibly due to shorter treatment time compared to previous reports. Previous studies showed that the ability of 5-Aza-dC to enhance expression of *cyclin D2 *was more marked if gastric cancer cells were treated for at least 5 days [25].

## Discussion

Epigenetic silencing through promoter hypermethylation and histone deacetylation is responsible for transcriptional repression of numerous tumor suppressor genes and growth regulatory genes in cancer cells [32-34]. A tightly regulated balance exists in normal cells among these processes, but disruption of this balance contributes to carcinogenesis. Recent studies have suggested novel chemo-preventative properties of SFN as an HDAC and DNMT inhibitor [15,35]. SFN was shown to have anti-proliferative and pro-apoptotic effects in many cancer cells, including prostate cancer [35-37]. However, the molecular mechanisms by which SFN regulates various genes that govern these processes remain to be elucidated. The current study demonstrates that SFN also impacts global DNA methylation and site-specific de-methylation of the *cyclin D2 *promoter. These results suggest that the ability of SFN to alter DNA methylation of specific tumor suppressor genes may be an important mechanism leading to prostate chemoprevention.

Previous studies indicate that *cyclin D2 *promoter methylation is more extensive in malignant than nonmalignant human prostate tissue [31] and treatment with 5-Aza-dC and TSA increased *cyclin D2 *expression in prostate cancer cell lines [30]. In the same study, over-expression of *cyclin D2 *induced anti-proliferative effects on LnCap cells, but not PC3 cells, suggesting that the inhibitory effects of *cyclin D2 *is limited to AR-dependent prostate cancer cells [30]. Similar to previous reports, our study showed that LnCap cells had lower expression of *cyclin D2 *compared to BPH-1 cells, a non- tumorigenic cell line (data not shown). Previous studies have implicated that SFN is an HDAC inhibitor. In prostate cancer cells, such as LnCap and PC3 cells, SFN treatment significantly inhibits HDAC activity [35,38]. Combined with findings from these studies, SFN's anti-proliferative effects may be mediated, at least in part, through de-methylation of the *cyclin D2 *promoter and restoration of *cyclin D2 *expression. It is possible that SFN-mediated both HDAC and DNMT inhibition and collectively allows chromatin remodeling for access of various transcription factors and de-suppression of the *cyclin D2 *promoter.

To identify potential DNA methylation changes mediated by SFN, we examined the global methylation status and the methylation status of CpG islands in the *cyclin D2 *promoter region (-1700 to -1250) in LnCap cells. The methylation analysis indicated that 5-Aza-dC induced more systemic de-methylation by significantly decreasing global methylation, whereas SFN showed more pronounced de-methylating effects in the *cyclin D2 *promoter region. More specifically, there was a significant decrease in methylation at the transcription factor c-Myc binding region. C-Myc has been implicated in modulating gene expression through epigenetic modifications. Classically, c-Myc's repressive effects on gene expression are attributed to inhibition of Sp1 transcriptional activity [39], and *cyclin D2 *is directly induced by c-Myc [40]. More recently, it was suggested that c-Myc modulates DNMT activity through direct binding or indirectly through an interaction with Mitz-1 to repress transcriptional activity, such as observed with the p21 promoter [39,41,42]. Furthermore, the core consensus sequence of c-Myc DNA binding site (CACGTG) contains a CpG site which is frequently methylated *in vivo*. Methylation of the consensus site blocks Myc/Max binding and trans-activation, but whether this influences the dynamics of Myc/Max heterodimer interactions with chromosomes remains to be elucidated [43]. It is important to recognize that global de-methylation has also been linked to increase genomic instability, and future studies are crucial to investigate whether such effects are advantageous for chemo-prevention using de-methylating compounds such as SFN. Furthermore, it is unclear how SFN treatment leads to selective de-methylation of specific CpG sites, but SFN may indirectly affect regulations of DNMTs and their specificities toward certain CpG sites. Nevertheless, SFN's effect on de-methylating the c-Myc binding site suggests that restoration of transcriptional expression of *cyclin D2 *may be dependent on c-Myc trans-activation. Future experiments utilizing chromatin immunoprecipitation (ChIP) will be essential to elucidate the detailed interactions and the role of c-Myc binding. Furthermore, it is possible that other regions may demonstrate more pronounced differential methylation pattern with SFN treatment. The analysis of other regions on the promoter and their impact on *cyclin D2 *re-expression is an important area for future research. Moreover, the higher treatment concentration (30 μM) in this study that altered methylation pattern in the *cyclin D2 *promoter (Figure 4) was a somewhat high concentration compared to SFN derived from dietary sources. However, it is possible that longer exposure of SFN at lower dosages could achieve the same outcomes. The findings here provide a proof of concept of the effects of SFN on DNA methylation, but the precise dosing regimens and treatment timing to obtain optimal effects are important areas of research.

Consistent with previous findings that SFN decreased expression of *DNMT1 *and *DNMT3a *in breast cancer cells [15], SFN also decreased DNMTs expressions in our study. We found that *DNMT1 *and *3a *mRNA expressions were significantly decreased by SFN in BPH-1 and PC3 cells, but only a trend of decrease in *DNMT1 *protein expression. In contrast, we found that *DNMT1 *and *3b *were down-regulated with SFN treatment in LnCap cells, but not *DNMT3a*. These results suggest that the responses to SFN treatment are specific to cell types. SFN may act as a dual epigenetic regulator by inhibiting both HDACs and DNMTs, leading to altered gene expression and contribute to anti-proliferative effects. It has been well established that DNA methylation and histone modifications cross-talk and cooperate in the regulation of gene transcription. Recent *in vitro *studies have shown that *DNMT1 *interacts physically with HDAC1 or 2. DNMTs recruit class 1 HDACs to function as co-repressors in the transcription of tumor suppressor genes [44-46]. Jones et al. demonstrated that trichostatin A (TSA), a HDAC inhibitor, relieves transcriptional repression at methylated CpG islands, and implicated that DNA methylation provided the nucleosomal infrastructure for HDAC-dependent chromatin modification and transcriptional silencing [34,47]. The SFN-mediated inhibition of DNMT expressions may be an important contributing factor in facilitating de-methylation of *cyclin D2 *promoter observed in this study. In addition to transcriptional regulation of DNMTs, it is also possible that alternative post-transcriptional and post-translational regulations of DNMTs are targeted by SFN. SFN may modulate histone profile and DNMT pathways at the same time. Previous studies from our lab established that SFN (≥ 15 μM) significantly decreased HDAC activity and expressions in LnCap cells [38,48]. SFN also potently increased p21 expressions. *DNMT1 *has been shown to competitively bind to proliferating nuclear cell antigen (PCNA) at the same site as p21, a tumor suppressor that inhibits DNA replication [49]. In ovarian cancer cells, SFN inhibits degradation of retinoblastoma protein (Rb), which directly binds to *DNMT1*, sequesters its activity and disrupts formation of DNMT-DNA complexes [46,50]. It is very likely that SFN regulates DNMT indirectly through HDACs or other protein interactions. Further examination of the direct coordination between these epigenetic marks is an exciting area for future studies. Studies that identify specific methyl binding proteins, transcriptional activators or repressors and chromatin remodeling will be essential to decipher the exact mechanism of *cyclin D2 *promoter regulation by SFN. Furthermore, SFN also decreased telomerase reverse transcriptase (*hTERT*) expression through promoter de-methylation and epigenetic modulations in breast cancer cells, suggesting that SFN may target other hypermethylated gene promoters that are dys-regulated during carcinogenesis and warrant further investigation. Overall, we demonstrated the SFN induced up-regulation of *cyclin D2 *in prostate cancer cells, and examined SFN as an epigenetic modulator by altering methylation status in the *cyclin D2 *promoter region. These findings provide additional insights into the mechanisms by which SFN may act as a diet-derived epigenetic modulator of gene expression and agent for prostate cancer prevention.

## Competing interests

The authors declare that they have no competing interests.

## Authors' contributions

AH performed the experiments including qRT-PCR for DNMTs and *Cyclin D2*, the global methylation analysis, bisulfite sequencing, and data and statistical analysis. YZ performed the immunoblot analysis on DNMTs, and CW performed the MSP. AH, RD, DW and EH conceived and participated in design and coordination of the study. AH and EH drafted the manuscript. All authors read and approved the final manuscript.
